# BRAF V600E mutation and high expression of PD-L1 in Rosai-Dorfman disease: case report and review of the literature

**DOI:** 10.1007/s12308-024-00611-9

**Published:** 2024-11-27

**Authors:** Farnoush M. Moen, Mariam M. Youssef, Mihir Shukla, Mary Lynn Nierodzik, Marius E. Mayerhoefer, Christopher Park

**Affiliations:** 1 Department of Pathology, NYU Langone Health, NYU Grossman School of Medicine, New York, NY USA; 2 Department of Pathology and Laboratory Medicine, UC Davis, Davis, CA USA; 3 Department of Medicine, NYU Langone Health, NYU Grossman School of Medicine, New York, NY USA; 4 Department of Radiology, NYU Langone Health, NYU Grossman School of Medicine, New York, NY USA

**Keywords:** Histiocytosis, Histiocytic/dendritic cell neoplasms, Rosai-Dorfman disease, Erdheim-Chester disease, BRAF V600E mutation, BRAF inhibitors, PDL1 inhibitors, Checkpoint blockade inhibitors, MAPK pathway

## Abstract

BRAF V600E mutations are frequently found in histiocytic/dendritic cell neoplasms such as Erdheim-Chester disease (ECD) and Langerhans cell histiocytosis (LCH), but few reports have also described BRAF mutations in Rosai-Dorfman disease (RDD), and even these cases may predominantly represent mixed histiocytosis. BRAF mutations have been studied in histiocytic/dendritic cell neoplasms and described to be associated with increased risk of relapse and long-term consequences, but few studies have examined BRAF V600E mutation in RDD, which is recognized as a neoplasm given the high frequency of MAPK pathway alterations. Here, we report a case of BRAF V600E-mutated RDD in a patient who presented with generalized lymphadenopathy. During our evaluation of this patient, we also found expression of PD-L1 in neoplastic histiocytes. During our review period, only few cases of RDD reported to harbor BRAF mutation or were evaluated for the expression of PDL1 by neoplastic cells. Given the potential challenges in distinguishing RDD from other histiocytic/dendritic cell neoplasms, including mixed histiocytosis with similar clinicopathological manifestations, we will discuss the current state of knowledge regarding the frequency and clinical impact of BRAF V600E in RDD, as well as the role of BRAF mutations in RDD pathogenesis. Distinction of BRAF V600E mutated histiocytic/dendritic cell neoplasms requires consideration of distinctive histopathological and immunophenotypic findings in appropriate clinical and radiologic setting. Given the increasing use of BRAF inhibitors as well as checkpoint blockade inhibitors to treat a number of cancers, we will discuss the clinical implications of the presence of BRAF V600E mutation and PD-L1 expression in RDD.

## Introduction

The histiocytoses represent a heterogeneous group of abnormal proliferations of mature histiocytic/dendritic cells. In the 2016 revised classification of histiocytoses, Rosai-Dorfman disease (RDD, also known as massive lymphadenopathy with sinus histiocytosis), was placed in the “R” group, which can be distinguished from “L” group (Langerhans) based on the absence of CD1a and CD207 expression. RDD can be subclassified based on clinical features and includes a familial type due to FAS deficiency, classic (nodal), extranodal, immune disease-associated, or neoplasia-associated forms that occur post-leukemia or post-lymphoma, or in association with other histiocytic neoplasms [1]. Recent studies have identified mutations involving the MAPK/ERK pathway in 30–50% of RDD cases, supporting the neoplastic nature of RDD histiocytes [2–4]. The 5th edition of the WHO Classification of Hematolymphoid Tumors subcategorizes the dendritic cell neoplasms under the spectrum of histiocytic/dendritic cell neoplasms based on their histogenesis and their distinct immunophenotypic profile (positive for CD45, CD4, CD68, CD163, and lysozyme). Thus, RDD joins histiocytic sarcoma (HS), Erdheim-Chester disease (ECD), juvenile xanthogranuloma (JXG), and the newly described ALK + histiocytosis in this category [5].

RDD is characterized by accumulation of large histiocytes in various organs resulting in variable clinical presentations and behavior. Specific features can allow discrimination of RDD from other histocytoses. Histologic features supportive of RDD and ECD include a proliferation of bland histiocytes with foamy cytoplasm. Unlike Touton giant cells which are common in JX, ALK + histiocytosis, and ECD [6, 7], they are not typical of RDD. Emperipolesis is a biological process in which inflammatory cells enter another living cell but remain intact, which is distinct from phagocytosis, where the internalized cell is killed. Emperipolesis is a frequent histologic feature in RDD, although it also has been reported in ECD and HS [8–11]. The neoplastic histiocytes in RDD express the histiocytic markers CD163 and CD68, as well as S100, but this immunophenotype is not completely unique to RDD. Assessment of BRAF mutational status has been used to aid in the diagnosis and guide treatment of histiocytic/dendritic cell neoplasms. BRAF V600E is frequently observed in LCH and ECD, but it has been reported in only a few cases of RDD [4, 12–16], with two previous studies of 23 and 21 RDD patients failing to identify BRAF V600E positive cases [3, 17]. Patients with classical RDD present with bilateral cervical lymphadenopathy, but 43% of patients with RDD present with extranodal disease [2]. Overall, while a diagnosis of RDD can frequently be made on the basis of histologic and immunophenotyping findings, in cases where the findings overlap with other histiocytic/dendritic cell neoplasms, a complete review of the clinical presentation is essential for accurate diagnosis.

## Case presentation

A 35-year-old male developed a right-sided neck mass with subsequent ultrasound showing prominent right-sided lymph nodes, including a 2.2 × 1.4-cm submandibular lymph node. These findings occurred in the context of fatigue, night sweats, and weight loss over the previous 6–8 weeks. CT of the chest without contrast showed a 1.4-cm left axillary lymph node as well as a right T8 body lesion with mild adjacent soft tissue thickening. CT of the abdomen/pelvis without contrast identified lymph nodes in the gastro-hepatic ligament and peri-gastric areas, with a mildly prominent right inguinal lymph node. On the CT images, there was no evidence of intracranial mass, abnormal enhancement, midline shift, or significant ventricular dilatation. Tests for HIV, EBV, CMV, toxoplasma, and tuberculosis were negative. MRI of the thoracic spine with and without contrast showed a focal defect at the right T8 superior endplate with extension into the paravertebral soft tissues.

**Fig. 1:**
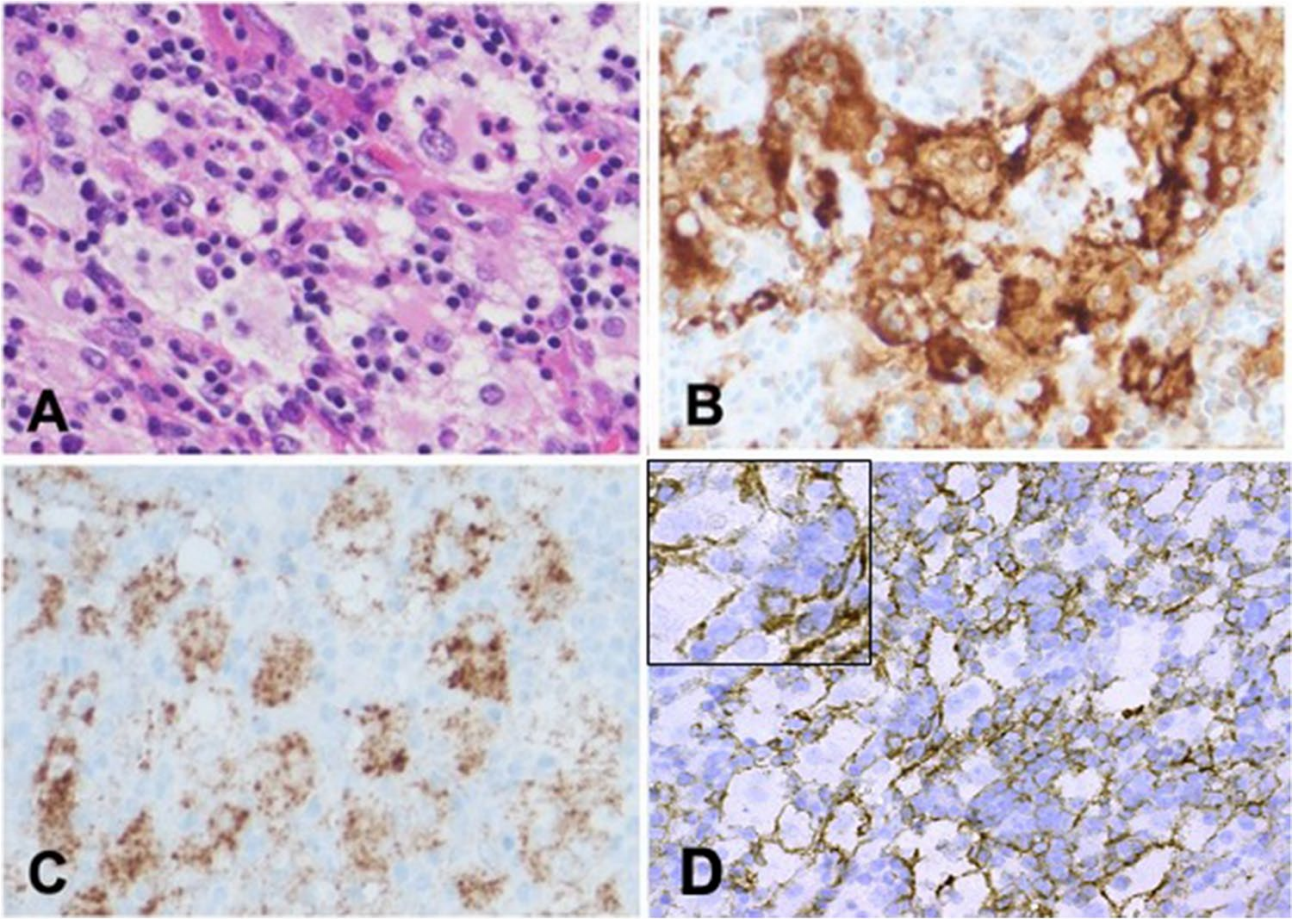
Histopathological and immunohistochemistry characteristics of the case. **A** H&E stain showing histiocytes with emperipolesis. **B** Immunohistochemistry analysis revealing atypical histiocytes positive for CD163. **C** Immunohistochemistry with VE1 showing atypical histiocytes containing the mutant BRAF V600E protein. **D** RDD histiocyte (inset) showing PD-L1 membranous staining (2 +) observed in 25–30% of histiocytes

**Fig. 2:**
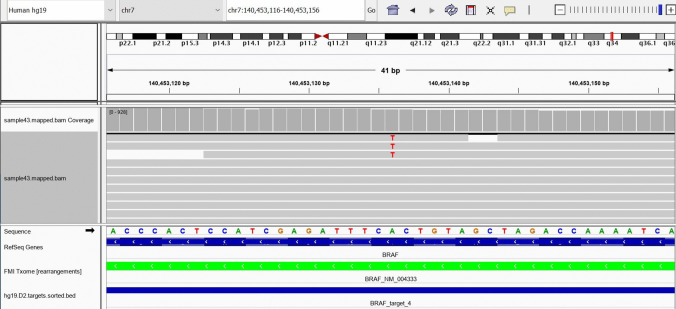
Foundation Medicine NGS sequencing data showing the presence of a BRAF (NM_030621.3) variant: p V600E (c.1799 T > A) at 1.7% variant allele frequency

The patient underwent a right cervical excisional lymph node biopsy. Sections of the lymph node showed effacement of normal architecture by an atypical histiocytic infiltrate admixed with many plasma cells. The atypical histiocytes were large in size with round, hypochromatic nuclei—some with conspicuous nucleoli—and ample cytoplasm. Numerous histiocytes contained lymphocytes and neutrophils compatible with emperipolesis. Immunohistochemical stains showed that the atypical histiocytes were positive for CD163 (MRQ-26), S-100 (4C4.9), BRAF (V600E-VE1), and Cyclin-D1 (SP4-R) and negative for CD1a (EP3622), CD23 (SP23), and ALK-1(ALK01). MUM1 (MRQ-43) and CD138 (B-A38) (Fig. 1) highlighted numerous plasma cells in the background. Immunohistochemical stains were scored as negative, or positive if present in at least 30% of RDD macrophages with weak (1 +), intermediate (2 +), or strong (3 +) staining. Using the FDA-cleared PD-L1 IHC 22C3 PharmDx kit and Dako Autostainer (Gen Path Oncology), tumor cells showed 2 + membranous staining in 25–30% of neoplastic histiocytes, which was confirmed by three independent hematopathologists. Foundation Medicine NGS sequencing data showed the presence of a BRAF (NM_030621.3) variant: p V600E (c.1799 T > A) at a variant allele frequency of 1.7% (Fig. 2).

The patient subsequently underwent 18F-FDG PET/CT (including contrast-enhanced companion CT) which showed hypermetabolic activity in the bilateral internal jugular and right submandibular and left axillary lymph nodes as well as a hypermetabolic lytic lesion in the right T8 vertebral body with adjacent right paraspinal soft tissue hypermetabolic activity. Similarly, none of the classic findings associated with Erdheim-Chester disease, such as sclerotic osseous lesions with/without hypermetabolism, cardiac pseudotumor, pulmonary septal thickening or cysts, perirenal infiltration, or brain lesions [18], was observed. The patient was admitted for right inguinal pain with a testicular ultrasound showing an abnormal right inguinal lymph node. MRI of the thoracic spine with/without contrast showed enhancement of the T8 vertebral body bone marrow edema pattern with associated cortical erosion, with enhancing soft tissue extension into prevertebral space. After discharge, he underwent CT-guided biopsy of the thoracic spine which showed fragments of sclerotic bone and scant, crushed marrow. Immunostaining showed rare possible bone marrow myeloid, erythroid, and lymphoid elements. CD163 immunostain showed few possible histiocytes. CD1a and S-100 were negative. Due to the scant histiocytes l, BRAF immunostaining was not performed, and no molecular testing was performed due to decalcification in Formical-4 solution. Additionally, secondary expert opinion on the material reported sclerotic bone, rare hematopoietic elements, and no atypical cells. Subsequently, the patient was managed supportively. The patient later presented with right neck swelling. CT of the neck showed right-sided lymphadenopathy, resulting in a right neck lymph node conglomerate/mass excisional biopsy. Sections from this biopsy showed histologic features similar to the prior right cervical lymph node biopsy. Repeat PET/CT after continued supportive management revealed resolution of hypermetabolic activity in the lymph nodes in the neck, supraclavicular fossae, left axilla, and right groin, as well as an interval decrease in the size and intensity of the hypermetabolic lytic lesion in the T8 vertebral body, and decreased intensity of the hypermetabolic activity in the paraspinal soft tissue adjacent to the right T8 vertebral body.

## Discussion

BRAF V600E mutations are present in tumors of histiocytic/dendritic cell origin with the highest frequency in LCH and ECD among histiocytic/dendritic cell neoplasms. The frequency of BRAF mutation in ECD in the lesion and peripheral blood monocytes has been reported to be between 51 and 67% [17, 19, 20]. Most large series recognize BRAF V600E mutation in 57% of LCH [21]. BRAF mutations have been reported in other histiocytic/dendritic cell neoplasms, but some may represent mixed histiocytosis. JXG has been reported to harbor BRAF mutation both in the CNS and ocular lesions [22–24]. Out of 10 pediatric CNS-JXG, 5 showed a BRAF mutation. However, after clinico-radiographic correlation, only 3 showed localized disease and others with systemic disease included pediatric ECD [24]. Most large studies report recurrent activating genetic alterations involving the classical MAPK signaling pathway in 57% of HS, but only a few reports either BRAFV600E or other BRAF mutations in HS [25, 26]. RDD has also been reported to show MAPK alterations in 30–50% of cases; however, no BRAF mutants were identified in RDD patients even in large studies [3, 27].

Recently, a few cases of RDD have been reported to harbor BRAF V600E mutations (Table 1). BRAF V600E has been reported in nodal RDD presenting with progressive bilateral cervical lymphadenopathy that improved without therapy [12]. A BRAF mutant RDD patient who presented with CNS involvement experienced treatment failure with steroids and died [13]. Recently, a patient with BRAF positive RDD who presented with CNS involvement responded to BRAF/MEK inhibitors [14]. RDD patients with BRAFY472C and BRAFR188G variants have been reported, but additional clinical features were not described [15]. A report of mixed RDD/LCH with BRAF mutation presented with diffuse lymphadenopathy, bone lesions, cerebellar lesions, and diagnostic RDD/LCH lesions in the lymph node and LCH lesion in the cerebellum [16]. Thus, to our knowledge, our case is only the second reported case of BRAF V660E mutated RDD associated with typical histologic and clinical findings.

**Table 1:** Reported BRAF-Mutated RDD or mixed histiocytosis with emperipolesis

Ref	Age (years)	Gender	Clinical picture	Histopathological examination	Mutation	Treatment	Diagnosis
The current case	34	Male	Lymphadenopathy T8 lesion	Emperipolesis CD163 + /S100 + /BRAF + /CyclinD1 + / PDL1 score: 2 +	BRAF V600E Next Gene Sequencing (Foundation 1)	Supportive Interval improvement of imaging findings	RDD
Fatobene et al. (2018) [12]	18	Male	Cervical Lymphadenopathy	Emperipolesis CD68 + /S-100 + /CD1a- VE1 +	BRAF V600E Multiplex picodroplet digital PCR	Resolved without treatment	RDD
Cronin et al. (2022) [14]	12	Male	Short stature hypopituitarism Progressive large suprasellar mass	Emperipolesis Fibrotic stroma KP + /CD68 + /S-100 + / CD1a-	BRAF V600E Cobas 4800 test assay	Failed on steroids, progressed on radiation, responded to BRAF-inhibitor therapy	RDD
Richardson et al. (2018) [13]	64	Female	Sixth nerve palsy Multiple enhancing region in brain stem	Diagnosis at autopsy Emperipolesis	BRAF exon12 Sanger Sequencing	Died	RDD
Dufour et al. (2021) [28]	Not provided	Not provided	Not provided	Not provided	BRAFY472C and BRAFR188G	Not provided	RDD
Mastropolo et al. (2019) [16]	6	Not provided	Lymphadenopathy Bone lesion in maxilla and C4, iliac and cerebellum, basal ganglia and corpus callosum	Lymph node: Emperipolesis histiocytes (S-100 + /Fascin + /BRAF (VE1) +) intermixed with LCH-like histiocytes (S-100 + /CD1a + /focal Fascin /BRAF (VE1) +) Cerebellum: LCH	BRAF V600E in PBMCs	Cytarabine followed by Dabrafenib with persistent disease added MEK inhibitor trametinib Follow up not provided	RDD/LCH

Although histiocytic/dendritic cell neoplasms are recognized by virtue of their histogenesis, some arise following the acquisition of somatic mutations in hematopoietic stem/progenitor cells (HSPCs). Xenotransplantation of CD34 + HSPCs from BRAF mutant patients with histiocytosis (ECD or MH) resulted in the development of histiocytosis-like lesions and detection of BRAF V600E in circulating monocytes of ECD and mixed histiocytosis patients. In addition, BRAF mutations were identified in CD34 + HSPCs in a subset of the same patients [29]. Studies of LCH patients identified BRAF mutations in HSPCs including common myeloid progenitors (CMP) and granulocyte–macrophage progenitors (GMP) [30]. Studies in LCH have shown BRAF mutations in the peripheral blood and CD34 + HSPCs of high-risk multisystem disease patients, while the presence of a BRAF mutation only in lesional tissue correlated with low-risk disease with a possible increased risk of recurrence [31, 32]. Given that HSPCs are the disease-initiating cells in ECD and LCH, it is thus not surprising that myeloid neoplasms develop in approximately 10% of LCH and ECD patients [33]. It will be interesting to evaluate both HSPCs and lesional cells in RDD patients to assess for the presence of BRAF mutations as well as correlate the pattern of BRAF mutation involvement with clinical outcomes, especially the development of myeloid neoplasms.

Given their overlapping morphologic and immunophenotypic features, the diagnostic approach to histiocytic/dendritic cell neoplasms should include a thorough review of clinical history. RDD usually presents as nodal disease but can be extranodal or multisystemic in 43% and 19% of cases, respectively. Cutaneous or CNS involvement can also be seen infrequently. Bone involvement occurs in 5–10% of RDD cases, typically in association with nodal disease, similar to this case. Bone lesions in RDD typically occur in the metaphysis or diaphysis and are osteolytic or mixed lytic/sclerotic [2]. Therefore, lesions in the femurs and tibia should raise concern for ECD since bone lesions in ECC are nearly always present as symmetric diaphyseal and metaphyseal osteosclerosis in the distal ends of the femurs and the proximal and distal tibia. Imaging studies are helpful, as ECD is frequently associated with dense infiltration of perinephric fat based (“hairy kidney”) as well as circumferential soft-tissue sheathing of the thoracic and abdominal aorta and its branches (“coated aorta”) [34]. Therefore, patients with similar histologic features presenting with single or multisystem lymphadenopathy most likely have RDD (similar to the patient in our case), while those with bone lesions and retroperitoneal or vascular findings without nodal involvement likely have ECD.

Cases of mixed histiocytosis that include both ECD and RDD components are difficult to ascertain since they both share similar histologic and immunophenotypic features, suggesting that at least some cases of RDD may have similar pathogenesis to ECD. Given the wide variety of presentations, it is unclear whether RDD represents a single disease. Emperipolesis can be associated with ECD and more frequently with MAP2K mutation. ECD with RDD-like lesions presenting as intra-abdominal and retroperitoneal lesions attributed to MAP2K1 mutation similar to three cohorts in which the frequency of RDD histology in ECD was reported as 3.6% (6/168), 3.1% (3/96), and 2.2%% (2/89) in overlap cases with typical clinical findings of bone lesions and/or retroperitoneal findings and/or coated vessels on imaging studies. In one cohort, 9 of 13 cases harbored MAP2K1 mutations, but none had BRAF mutations [10, 11]. Cases with CNS or cardiac involvement are particularly problematic. BRAF mutations have been described in a case characterized by CNS involvement with histologic evidence of emperipolesis recognized as ECD/RDD overlap disease [28], as well as in a case presenting with multiple bone lesions and pericardial effusion reported as ECD with emperipolesis [35].

RDD has an unpredictable clinical course and is fatal in 5–10% of cases, and thus, MAPK/RAF inhibitors have been used to treat RDD given the frequency of MEK pathway mutations. Cournoyer et al. treated 34 histiocytosis with favorable response in 94% of cases receiving either dabrafenib and/or trametinib after failure of chemotherapy and all cases receiving the inhibitor as first-line treatments. However, only 1 of 2 RDD patients with no identifiable mutation responded [36]. In a study of 16 RDD cases including KRAS- and MEK-variant cases, treatment with cobimetinib was associated with 88% vs. 38% overall response rates in mutant vs. wild-type RDD [37]. These studies led to FDA-approval of cobimetinib for the treatment of RDD and MEK inhibitors, which are essentially considered the first line of therapy for such patients [38]. Immunotherapy is usually not necessary in RDD cases since their clinical behavior is akin to a low-grade indolent histiocytic neoplasm. However, PD-L1 expression by cases of atypical cases of RDD exhibiting more aggressive behavior might justify treatment with immunotherapy, although this would require more clinical studies.

Given that vemurafenib induces responses in BRAF mutant ECD or LCH at high rates [39–41], it is not surprising that BRAF or MAPK inhibitor therapy has been described in BRAF mutant RDD. Cronin et al. treated one patient with a BRAF-mutated RDD CNS tumor [14], while Mastropolo et al. treated a case of BRAF V600E RDD and LCH with cytarabine, then dabrafenib. There was no response to either agent, and therefore, trametinib was added for concurrent MEK inhibition [16]. Diamond et al. found in their phase II trial that 18 patients with histiocytic neoplasms treated with cobimetinib had an 89% overall response rate; this trial included two patients with RDD, although neither harbored BRAF mutations [42].

The PD1/PD-L1 pathway regulates the balance between the stimulatory and inhibitory signals that regulate T cell responses needed for immune defense and tolerance. Ligation of PD-1 by PD-L1 activates a critical immune checkpoint leading to T cell dysfunction, exhaustion, and tolerance. High-affinity anti-PD-1 or anti-PD-L1 monoclonal antibodies (mAbs), which block their interactions, can reverse the immune checkpoint, releasing the brake on T cell responses [43]. PD-L1/PD-L2 can be expressed by normal or tumor-associated macrophages/ histiocytes. Neoplastic cells in histiocytic neoplasms also can express PD-L1, but checkpoint protein expression in RDD is variable, with studies reporting positivity in 2/11 (18%) [44], 0/4 [45], or 16/28 (57%) of cases [46]. It should be noted, however, that none of these studies evaluated BRAF mutant RDD. While immunotherapy would not be a typical therapeutic consideration in RDD since it typically behaves as low-grade indolent neoplasm and also responds to MEK inhibitors, it is intriguing to consider checkpoint blockade as a possible adjunct to MEK or BRAF inhibitor therapy in more severe cases of RDD.

## Conclusion

Histiocytic/dendritic cell neoplasms are diagnosed by identifying distinctive histopathological and immunophenotypic findings in the appropriate clinical and radiologic context. Herein, we report a case of RDD that presented with clinical and histologic features that are typical for RDD. However, in distinction to most reported cases of RDD, the neoplastic histiocytes were positive for the BRAF V600E mutation. Since cases of mixed histiocytosis that include both ECD and RDD components do occur, we considered whether this lesion represents such a case. Although it is difficult to completely rule out this possibility since not all sites were sampled in this patient, we do not think the disease represents ECD given the clinical presentation, available histologic data, FDG PET/CT Scan [18], and self-limited disease course.

RDD patients may exhibit variable clinical courses ranging from self-limited disease to death. Prognosis correlates with the number of nodal groups and extranodal systems involved, with CNS and multiorgan involvement associated with worse outcomes [47–49]. Our case was self-limited, suggesting that only lesional cells harbored the BRAF mutation. BRAF inhibitors may be considered in BRAF-mutated RDD in symptomatic cases. However, this needs to be studied. Therapeutic use of PD-1/PD-L1 blockade in histiocytic/dendritic cell neoplasms has been reported in cases of HS, with patients responding to nivolumab at progression [50, 51]; however, given the extremely limited experience using PD-1/PD-L1 blockade in histiocytic/dendritic cell neoplasms including RDD as well as variability in PD-L1 expression as determined by immunostain alone [52], treatment of RDD with checkpoint inhibitors cannot be recommended at present

## References

[1] Emile JF et al (2016) Revised classification of histiocytoses and neoplasms of the macrophage-dendritic cell lineages. Blood 127(22):2672–2681 26966089 10.1182/blood-2016-01-690636 PMC5161007

[2] Abla O et al (2018) Consensus recommendations for the diagnosis and clinical management of Rosai-Dorfman-Destombes disease. Blood 131(26):2877–2890 29720485 10.1182/blood-2018-03-839753 PMC6024636

[3] Graces S et al (2017) Mutually exclusive recurrent KRAS and MAP2K1 mutations in Rosai-Dorfman disease. Mod Pathol 30(10):1367–1377 28664935 10.1038/modpathol.2017.55 PMC5837474

[4] Ravindran R et al (2023) How I diagnose Rosai-Dorfman disease. Am J Clin Pathol 160(1):1–10 37167084 10.1093/ajcp/aqad047

[5] Khoury, JD., et al. (2022) The 5th edition of the World Health Organization classification of haematolymphoid tumours myeloid and histiocytic/dendritic neoplasms. Leukemia. 36(7):1703–1719 10.1038/s41375-022-01613-1 PMC9252913 35732831

[6] Kashima J et al (2021) ALK-positive histiocytosis of the breast: a clinicopathologic study highlighting spindle cell histology. Am J Surg Pathol 45(3):347–355 32826530 10.1097/PAS.0000000000001567

[7] Ozkaya N et al (2018) The histopathology of Erdheim-Chester disease: a comprehensive review of a molecularly characterized cohort. Mod Pathol 31(4):581–597 29192649 10.1038/modpathol.2017.160 PMC6718953

[8] Congyang L et al (2011) Synchronous histiocytic sarcoma and diffuse large B cell lymphoma involving the stomach: a case report and review of the literature. Int J Hematol 93(2):247–252 21286876 10.1007/s12185-011-0773-3

[9] Zheng YY et al (2010) Histiocytic sarcoma: a clinicopathologic study of 6 cases. Zhonghua Bing Li Xue Za Zhi 39(2):79–83 20388371

[10] Portegys J et al (2023) Erdheim-Chester disease with Rosai-Dorfman-like lesions: treatment with methotrexate, anakinra and upadacitinib. RMD Open. 9(1):e002852 36693681 10.1136/rmdopen-2022-002852 PMC9884847

[11] Razanamahery J et al (2020) Erdheim-Chester disease with concomitant Rosai-Dorfman like lesions: a distinct entity mainly driven by MAP2K1. Haematologica 105(1):e5–e8 31123032 10.3324/haematol.2019.216937 PMC6939531

[12] Fatobene G et al (2018) BRAF V600E mutation detected in a case of Rosai-Dorfman disease. Haematologica 103(8):e377–e379 29748446 10.3324/haematol.2018.190934 PMC6068029

[13] Richardson TE et al (2018) BRAF mutation leading to central nervous system rosai-dorfman disease. Ann Neurol 84(1):147–152 30014527 10.1002/ana.25281

[14] Cronin C et al (2022) Case report: BRAF-inhibitor therapy in BRAF-mutated primary CNS tumours including one case of BRAF-mutated Rosai-Dorfman disease. Front Med (Lausanne) 9:1070828 36619621 10.3389/fmed.2022.1070828 PMC9813211

[15] Chen J et al (2022) Diverse kinase alterations and myeloid-associated mutations in adult histiocytosis. Leukemia 36(2):573–576 34611301 10.1038/s41375-021-01439-3

[16] Mastropolo R et al (2019) BRAF-V600E-mutated Rosai-Dorfman-Destombes disease and Langerhans cell histiocytosis with response to BRAF inhibitor. Blood Adv 3(12):1848–1853 31213430 10.1182/bloodadvances.2019000093 PMC6595264

[17] Haroche J et al (2012) High prevalence of BRAF V600E mutations in Erdheim-Chester disease but not in other non-Langerhans cell histiocytoses. Blood 120(13):2700–2703 22879539 10.1182/blood-2012-05-430140

[18] Hazim AZ et al (2022) Classical and non-classical phenotypes of Erdheim-Chester disease: correlating clinical, radiographic and genotypic findings. Br J Haematol 199(3):454–457 36017680 10.1111/bjh.18422

[19] Emile JF et al (2013) BRAF mutations in Erdheim-Chester disease. J Clin Oncol 31(3):398 23248255 10.1200/JCO.2012.46.9676

[20] Cangi MG et al (2015) BRAFV600E-mutation is invariably present and associated to oncogene-induced senescence in Erdheim-Chester disease. Ann Rheum Dis 74(8):1596–1602 24671772 10.1136/annrheumdis-2013-204924

[21] Badalian-Very G et al (2010) Recurrent BRAF mutations in Langerhans cell histiocytosis. Blood 116(11):1919–1923 20519626 10.1182/blood-2010-04-279083 PMC3173987

[22] Techavichit P et al (2017) BRAF V600E mutation in pediatric intracranial and cranial juvenile xanthogranuloma. Hum Pathol 69:118–122 28504206 10.1016/j.humpath.2017.04.026

[23] Zhang C et al (2021) Ocular juvenile xanthogranuloma with BRAF V600E mutation in a child. J Pediatr Ophthalmol Strabismus 58(4):e19–e21 34288768 10.3928/01913913-20210416-01

[24] Picarsic J et al (2019) BRAF V600E mutation in juvenile xanthogranuloma family neoplasms of the central nervous system (CNS-JXG): a revised diagnostic algorithm to include pediatric Erdheim-Chester disease. Acta Neuropathol Commun 7(1):168 31685033 10.1186/s40478-019-0811-6 PMC6827236

[25] Shanmugam V et al (2019) Identification of diverse activating mutations of the RAS-MAPK pathway in histiocytic sarcoma Mod Pathol. 32(6):830-843 10.1038/s41379-018-0200-x 30626916

[26] Shimono J et al (2017) Prognostic factors for histiocytic and dendritic cell neoplasms. Oncotarget 8(58):98723–98732 29228722 10.18632/oncotarget.21920 PMC5716762

[27] Zaman A, Wu W, Bivona TG (2019) Targeting oncogenic BRAF: past, present, and future. Cancers (Basel) 11(8):1197 31426419 10.3390/cancers11081197 PMC6721448

[28] Dufour J et al (2021) BRAF mutation in overlapping form of Erdheim-Chester and Rosai Dorfman diseases: a unique case restricted to the central nervous system. Rev Neurol (Paris) 177(6):708–710 33478735 10.1016/j.neurol.2020.09.010

[29] Durham BH et al (2017) Functional evidence for derivation of systemic histiocytic neoplasms from hematopoietic stem/progenitor cells. Blood 130(2):176–180 28566492 10.1182/blood-2016-12-757377 PMC5510787

[30] Milne P et al (2017) Hematopoietic origin of Langerhans cell histiocytosis and Erdheim-Chester disease in adults. Blood 130(2):167–175 28512190 10.1182/blood-2016-12-757823 PMC5524529

[31] Berres ML et al (2014) BRAF-V600E expression in precursor versus differentiated dendritic cells defines clinically distinct LCH risk groups. J Exp Med 211(4):669–683 24638167 10.1084/jem.20130977 PMC3978272

[32] Rafiei A et al (2020) BRAFV 600E or mutant MAP2K1 human CD34+ cells establish Langerhans cell-like histiocytosis in immune-deficient mice. Blood Adv 4(19):4912–4917 33035332 10.1182/bloodadvances.2020001926 PMC7556147

[33] Papo M et al (2017) High prevalence of myeloid neoplasms in adults with non-Langerhans cell histiocytosis. Blood 130(8):1007–1013 28679734 10.1182/blood-2017-01-761718 PMC5570678

[34] Diamond EL et al (2014) Consensus guidelines for the diagnosis and clinical management of Erdheim-Chester disease. Blood 124(4):483–492 24850756 10.1182/blood-2014-03-561381 PMC4110656

[35] Zhu P et al (2017) Erdheim-Chester disease with emperipolesis: a unique case involving the heart. Cancer Res Treat 49(2):553–558 27488869 10.4143/crt.2016.078 PMC5398381

[36] Cournoyer E et al (2024) Dabrafenib and trametinib in Langerhans cell histiocytosis and other histiocytic disorders. Haematologica 109(4):1137–1148 37731389 10.3324/haematol.2023.283295 PMC10985423

[37] Abeykoon JP, Rech KL, Young JR et al (2022) Outcomes after treatment with cobimetinib in patients with Rosai-Dorfman disease based on KRAS and MEK alteration status. JAMA Oncol 8:1816–1820 36201194 10.1001/jamaoncol.2022.4432 PMC9539729

[38] American Society of Clinical Oncology. (2023) The ASCO Post: FDA approves oral MEK inhibitor cobimetinib for histiocytic neoplasms. 2022. https://ascopost.com/issues/december-10-2022/fda-approves-oral-mek-inhibitor-cobimetinib-for-histiocytic-neoplasms/. Accessed 17 Apr2023

[39] Hyman DM et al (2015) Vemuraranefib in multiple non melanoma cancers with BRAF V600 mutation. N Eng J Med 373(8):726–36 10.1056/NEJMoa1502309 PMC4971773 26287849

[40] Oneal PA et al (2018) FDA approval summary: vemurafenib for the treatment of patients with Erdheim-Chester disease with the BRAFV600 mutation. Oncologist 23(12):1520–1524 30120160 10.1634/theoncologist.2018-0295 PMC6292556

[41] Diamond EL et al (2018) Vemurafenib for BRAF V600-mutant Erdheim-Chester disease and Langerhans cell histiocytosis: analysis of data from the histology-independent, phase 2, open-label VE-BASKET study. JAMA Oncol 4(3):384–388 29188284 10.1001/jamaoncol.2017.5029 PMC5844839

[42] Diamond EL et al (2019) Efficacy of MEK inhibition in patients with histiocytic neoplasms. Nature 567(7749):521–524 30867592 10.1038/s41586-019-1012-y PMC6438729

[43] Keir ME, Francisco LM, Sharpe AH (2007) PD-1 and its ligands in T-cell immunity. Curr Opin Immunol 19(3):309–314 17433872 10.1016/j.coi.2007.04.012

[44] Xu J et al (2016) Expression of programmed cell death 1 ligands (PD-L1 and PD-L2) in histiocytic and dendritic cell disorders. Am J Surg Pathol 40(4):443–453 26752545 10.1097/PAS.0000000000000590

[45] Gatalica Z et al (2015) Disseminated histiocytoses biomarkers beyond BRAFV600E: frequent expression of PD-L1. Oncotarget 6(23):19819–19825 26110571 10.18632/oncotarget.4378 PMC4637323

[46] Ravindran R et al (2021) Rosai-Dorfman disease displays a unique monocyte-macrophage phenotype characterized by expression of OCT2. Am J Surg Pathol 45(1):35–44 33177341 10.1097/PAS.0000000000001617

[47] Chen J et al (2011) Rosai-Dorfman disease of multiple organs, including the epicardium: an unusual case with poor prognosis. Heart Lung 40(2):168–171 20561887 10.1016/j.hrtlng.2009.12.006

[48] Imada H et al (2015) A lethal intracranial Rosai-Dorfman disease of the brainstem diagnosed at autopsy. Pathol Int 65(10):549–553 26184902 10.1111/pin.12331

[49] Sendrasoa FA et al (2016) Rosai-Dorfman disease involving multiple organs: an unusual case with poor prognosis. Case Rep Med 2016:3920516 27872644 10.1155/2016/3920516 PMC5107849

[50] Imataki O et al (2022) Application of PD-L1 blockade in refractory histiocytic sarcoma: a case report. Mol Clin Oncol 17(3):136 35949894 10.3892/mco.2022.2569 PMC9353867

[51] Bose S et al (2019) Favorable response to nivolumab in a young adult patient with metastatic histiocytic sarcoma. Pediatr Blood Cancer 66(1):e27491 30270506 10.1002/pbc.27491 PMC6433376

[52] Jöhrens K (2021) The challenge to the pathologist of PD-L1 expression in tumor cells of non-small-cell lung cancer-an overview. Curr Oncol 28(6):5227–5239 34940076 10.3390/curroncol28060437 PMC8699902

